# A critical role of mir-199a in the cell biological behaviors of colorectal cancer

**DOI:** 10.1186/s13000-015-0260-x

**Published:** 2015-06-12

**Authors:** Hua Ye, Liping Pang, Qiong Wu, Yuzhen Zhu, Cancan Guo, Ying Deng, Xuebao Zheng

**Affiliations:** Guangdong Key Laboratory for Research and Development of Natural Drugs, Guangdong Medical College, Zhanjiang, Guangdong 524023 China

**Keywords:** MiR-199a, Colorectal cancer, Cell lines, *In vitro*, Hypoxia-inducible factor 1α, Vascular endothelial growth factor

## Abstract

**Background:**

Colorectal cancer (CRC) is one of the most common cancer and the leading causes of cancer mortality worldwide. The critical role of hypoxia-inducible factor 1α (HIF-1α) and vascular endothelial growth factor (VEGF) are important in the cancer development.

**Methods:**

The purpose of this study was to investigate the association of miR-199a expression in CRC and non-tumor tissues as well as assessed the effect of miR-199a on biological behaviors including cell proliferation, apoptosis, migration and invasion of CRC cells. The expression of miR-199a was distinctly decreased in colorectal cancer tissues compared with non-neoplastic colorectal tissues.

**Results:**

In this study, we found that miR-199a down-regulation was associated with the CRC and metastasis incidence. Advanced study showed that miR-199a up-regulation would lead to decreased CRC proliferation, migration and invasion. However, no significant association of miR-199a treatment and apoptosis rate and cell-cycle were detected in this study. The detection for the mechanisms of miR-199a on the development of CRC showed that the anticarcinogenic effect of miR-199a might be produced through HIF-1α/VEGF pathway.

**Conclusion:**

It was found that miR-199a would reduce the proliferation, migration and invasion. However, overexpression of miR-199a on the apoptosis rate and cell cycles showed no significant results. The potential functionary mechanism of miR-199a might through HIF-1α/VEGF pathway.

**Virtual slides:**

The virtual slide(s) for this article can be found here: http://www.diagnosticpathology.diagnomx.eu/vs/9806714131513041.

## Background

Colorectal cancer (CRC) is the third most common cancer and one of the leading causes of cancer mortality worldwide [1]. Despite considerable work on the tumourigenesis and progression of CRC, however, the detailed pathogenesis of this complex disease is poorly understood by now. The importance of hypoxia-inducible factor 1α (HIF-1α) in tumorigenesis is reported by the finding that HIF-1α was a potential important key regulatory factor in the development of CRC [2]. HIF-1α is overexpressed in a variety of tumors including CRC and the expression of HIF-α is often associated with poor prognosis [3,4]. In advance, HIF-1α is reported to be involved in kinds of progresses of tumorigenesis including proliferation, angiogenesis, metastasis, and chemotherapy resistance [5]. Besides, vascular endothelial growth factor (VEGF) is one of the most important downstream genes of HIF-1α. The critical role of VEGF in kinds of cancers has indicated that the depression of VEGF is an important method to treat certain cancers. Previous studies demonstrated that VEGF promotes various processes involved in angiogenesis, including endothelial cell proliferation, adhesion, migration, and chemotaxis [6]. Angiogenesis is a hallmark of cancer and has been targeted by various cancer therapies, with a focused effort on drugs that inhibit VEGF [7]. Advanced studies on the depression of the expression of HIF-1α and VEGF would provide better strategy for the treatment of CRC.

The microrna (MiRNA) is a class of single-stranded non-coding RNA with 18 to 25 nucleotides, and can regulate the relevant gene expression at post-transcriptional level through inhibiting degrading mRNA or protein translation of target gene [8]. There has been significant evidence showing that miRNAs regulate as many as more than 60% of human protein coding genes [9]. MiRNAs exert important functions in cancer development, cell differentiation, and regulation of cell cycles and apoptosis of cancer cells [10]. Nowadays a growing body of evidence indicated that miRNAs are key regulators that would contribute to the initiation and development of various types of cancer. In the development of cancer, normal regulatory mechanisms are disrupted by altered expression of tumor-suppressive or oncogenic miRNAs. Therefore identification of differentially expressed miRNAs during human oncogenesis would produce certain effects in the detection of physiopathological mechanisms of cancers. In a study by Kong *et al.*, it was reported that miR-199a was down-regulated in both CRC cell lines and tissues [11]. It was concluded that miR-199a can regulate CAC1 and reacted as a tumor suppressor in CRC. Besides, a recent study showed that miR-199a overexpression suppressed the hypoxia-induced proliferation of non-small cell lung cancer cells through targeting elevated HIF-1a and blocking the downstream upregulation of (Pyruvate dehydrogenase lipoamide kinase isozyme 1) PDK1 without affecting AKT activation [12]. Besides, another study showed that up-regulation of miR-199a suppressed cell proliferation, motility and angiogenesis of ectopic stem cells by targeting the 3′ untranslated region of VEGF [12]. In this study, we hypothesized that miR-199a could down-regulated both HIF-1 and VEGF and thus work as a CRC suppressor. The aim of the present study was to investigate the functional significance of miR-199a and to identify the molecular target genes regulated by miR-199a in CRC cells. Here, we investigated the expression of miR-199a in CRC. Besides, we assessed the effect of miR-199a on biological behaviours including cell proliferation, apoptosis, migration and invasion of CRC cells.

## Methods

### Tissue samples

Written informed consents were obtained from all the patients and all samples were collected according to the protocols approved by the Clinical Research Ethics Committee of Guangdong Medical College, Zhanjiang, Guangdong.

Tissue samples were prepared in a similar manner as described previously [13]. Briefly, 28 samples (14 tumor and 14 metastasis tissues from 15 males, 13 females; 59.18 ± 12.34 and 57.53 ± 11.21 years old, respectively) of CRC tissues were obtained from patients who underwent surgical resection at Guangdong Medical College, Zhanjiang, Guangdong 2013 to 2014. Nontumourous colorectal tissues more than 3 cm away from the tumors were selected as controls. None of the patients received preoperative treatment, such as radiation therapy or chemotherapy.

### RNA isolation

Total RNA was isolated using TRIzol reagent (Invitrogen, Carlsbad, CA, USA) according to the protocol of manufacturer. RNA concentrations were determined by spectrophotometer. RNA quality was confirmed using a NanoDrop 1000 Spectrophotometer (Thermo Fisher Scientific, USA).

### Quantitative real-time RT-PCR

Stem-loop RT-PCR (TaqMan microRNA assays; Applied Biosystems, Foster City, CA, USA) was used to quantify miRNAs according to manufacturer’s prtocal. To normalize the data for quantification of miR-199a, we used RNU48 as a reference. The ^∆∆^Ct method was used to calculate the fold-change.

### Cell transfection

Exponentially growing cells (1.5 × 10^5^) were seeded in 12-well plates 12 h before transfection and were transfected with 30 nM miR-199a precursor (miR-199a group) or negative control (control group) using the X-tremeGENE transfection reagent (Roche Applied Science, Indianapolis, IN, USA) according to the manufacturer’s instructions.

### Proliferation assay

Proliferation assay was conducted by the 3-(4,5-dimethylthiazol-2-yl)-2,5- diphenyl-2H-tetrazolium bromide (MTT) method. At 0 to 7 days post-transfection, the transfection medium in each well was replaced by 100 ml of fresh serum-free medium with 0.5 g/L MTT. After incubation at 37°C for 4 h, the MTT medium was removed, and 50 ml of Dimethyl Sulfoxide (DMSO) was added to each well. After incubation at 37°C for another 10 min, the A570 nm of each sample was measured using a plate reader.

### Apoptosis and cell-cycle analysis by flow cytometry

At 72 h post-transfection, the SW480 cells were harvested and re-suspended in phosphate-buffered saline (PBS) and then fixed in ethanol at −20°C overnight. The cells were washed with PBS and resuspended in staining solution (50 μg/ml propidium iodide, 1 mg/ml RNase A and 0.1% Triton X-100 in PBS). In brief, cells were trypsinized, collected and then stained using the Annexin V-FITC/PI Apoptosis Detection Kit following the manufacturer’s instructions. After incubation with Annexin V-FITC and PI, the apoptotic cells were immediately analyzed by flow cytometry. Early apoptotic cells were defined as the population that was PI negative and Annexin V-FITC positive, while late apoptotic cells were PI positive and Annexin V-FITC positive. The cell cycle detected by stained cells (1 × 10^5^) were analyzed with an FACScalibur; Becton Dickinson Flow Cytometer (PT. Madagasi Brosa Inc., Batang Hari, Propinsi Sumatera Utara, Indonesia).

### Western blot

Two groups of SW480 cells (miR-199a and control groups) were washed with ice-cold PBS for 3 times, added RIPA lysis buffer, lysed on ice for 30 min, scrapped off, transferred into EP tube and centrifuged at 4°C, 12000 × g for 30 min. The supernatants were collected and protein concentration was determined using BCA protein quantitation kit. Each sample with 60 μg protein was added 1 × SDS sample buffer, and denatured at 95°C for 5 min. Proteins were separated by 10% SDS-PAGE electrophoresis and transferred to 0.45 μm NC membrane. Membrane was blocked with 5% skim milk for 1 h, and incubated with 1:1000 diluted HIF-1aα and VEGF antibodies at 4°C overnight. The membrane was then incubated with 1:1000 diluted β-actin antibodies at room temperature for 1 h, washed with PBST three times, and detected with Odyssey system.

### Cell migration and invasion assays

SW480 cells was cultured in 25 cm^2^ culture flask to approximately 80%-90% density and then seeded into 6-well plates at 2.5 × 10^5^ cells/well. A 6-well plate was placed in cell culture incubator until cell monolayer reaches 40%-50% density. The transfected cells were incubated until the cell monolayer reached to 100% density. The bottom of 6-well plates was scratched with a P200 pipette tip and washed 3 times with PBS to wash off unattached cells. The width of scratch was observed at 48 h using inverted microscope (50-fold). Cell migration assay was conducted with transwell inserts with 8.0 mm pore size membrane (24-well format, Corning, New York, USA). To measure invasion ability of CRC cells, the previously mentioned inserts were pre-coated with matrigel matrix (BD Science, Sparks, MD, USA). The cells (1 × 10^5^) were re-suspended in serum-free medium and seeded to the upper chamber. The lower chambers were filled with complete culture medium containing 10% fetal calf serum (FBS). After incubation at 37°C for 24 h, the migrated cells present on the lower side of the membrane were fixed, stained and counted. Each experiment was performed in triplicate.

### Statistical analysis

Analyses were performed using the statistical package SPSS 19.0 (ver. 19.0; SPSS Institute Inc. Chicago, USA). Differences were analyzed with the Student’s *t*-test between two groups or with one-way ANOVA among three groups for the differences of miR-199a expression between cancer, metastasis and non-tumourous tissues. Data were expressed as the means ± standard deviation from at least 3 separate experiments. P values of < 0.05 are considered significant.

## Results

### MiR-199a down-regulation in CRC and metastasis tissues

**Figure 1:**
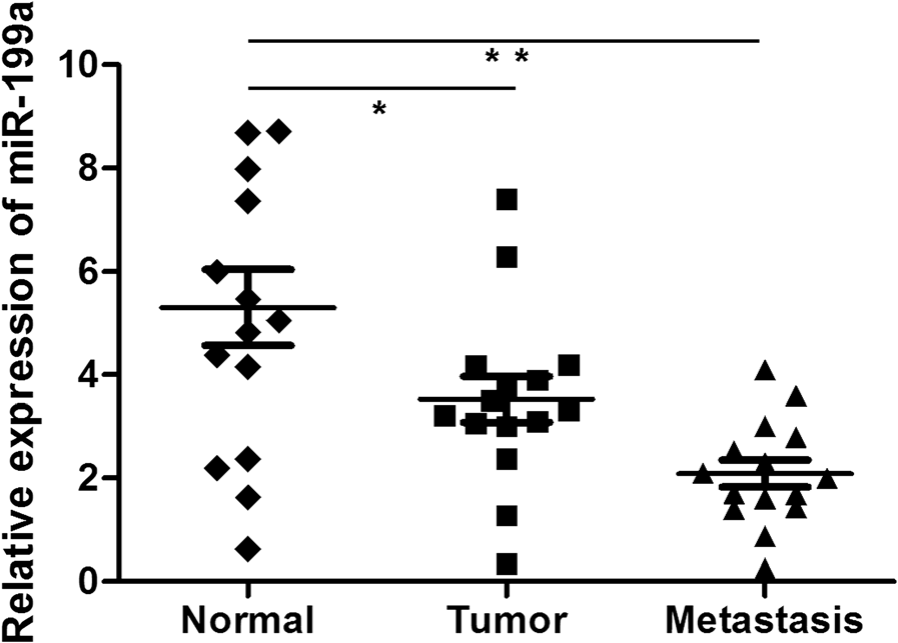
MiR-199a was significantly decreased in CRC tissues. The mRNA expression of miR-199a was measured by qRT-PCR in CRC, metastasis and adjacent normal tissue. Graph represents the 2^-∆∆Ct^ values ± SD, * P < 0.05; ** P < 0.01.

MiR-199a expression was detected in 14 CRC, 14 metastasis and adjacent non-neoplastic colorectal tissues normalized to RNU48. As shown in Figure 1, we found that the expression of miR-199a was distinctly decreased in colorectal cancer tissues compared with non-neoplastic lung tissues (mean ± SD: 3.7 ± 0.4 vs. 5.2 ± 0.8, P < 0.05). In addition, miR-199a expression in metastasis tissue was lower compared with the control group (mean ± SD: 2.2 ± 0.3 vs. 5.2 ± 0.8, P < 0.01).

### The effects of miR-199a overexpression on CRC cell growth

**Figure 2:**
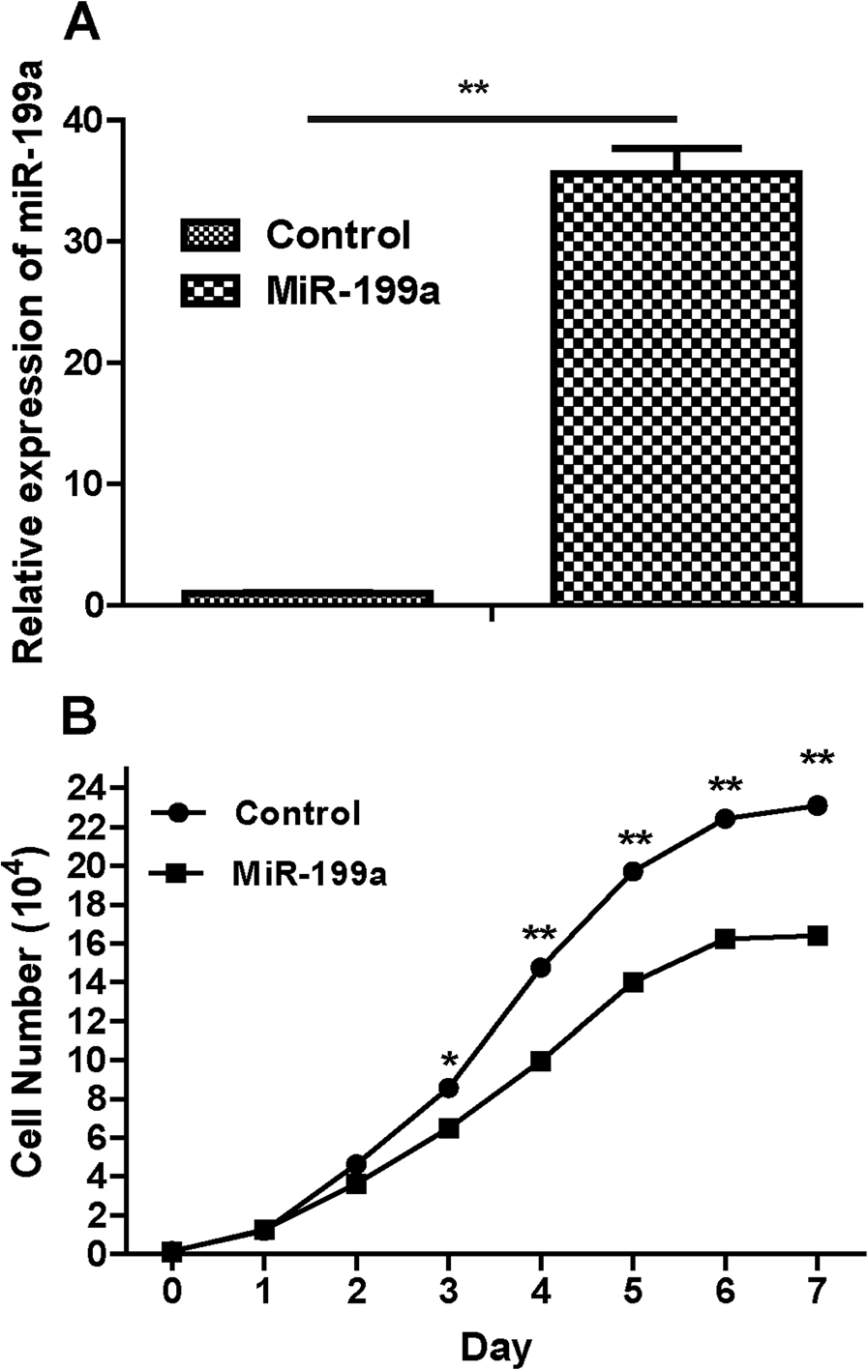
Overexpression of miR-199a on CRC cell growth. **(A)** Expression of miR-199a was determined in SW480 cells after miR-199a precursor transfection compared to controls. **(B)** The cell number of SW480 cells after miR-199a precursor transfection compared to controls.

In order to assess the effects of miR-199a on CRC cell growth, the miR-199a precursor was transfected into SW480 cells and cell growth at various post-transfection time points (0 to 7 days) was examined. As showed in Figure 2A, miR-199a precursor treatment was found to up-regulate miR-199a expression in SW480 cells. Besides, we found that miR-199a would reduce the cell growth after treated for more than 3 days (Figure 2B).

### The effects of miR-199a overexpression on CRC cell proliferation

**Figure 3:**
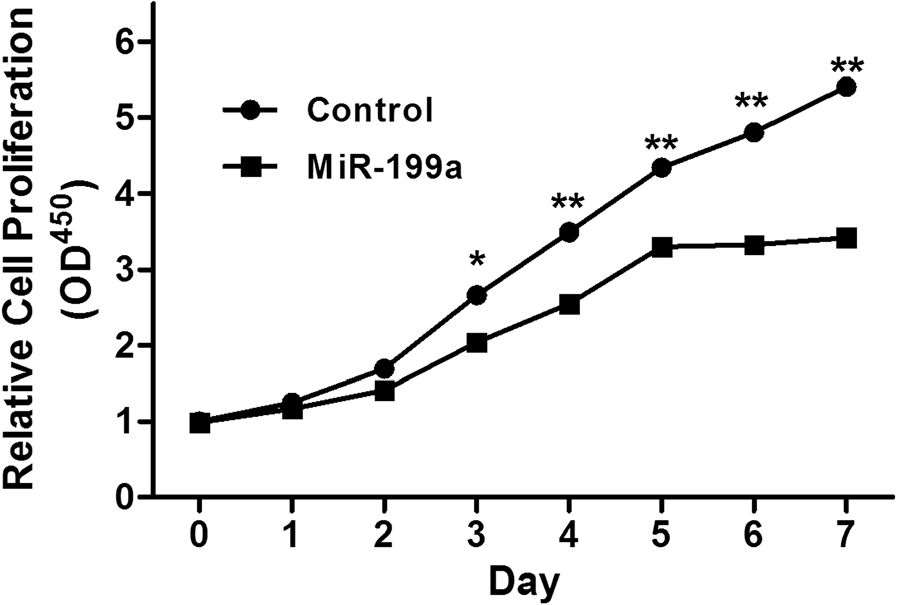
The cell proliferation was determined in SW480 cells transfected with miR-199a precursor or negative control (Ctrl). A_450_ absorption was assayed after transfection for 24 h.

As showed in Figure 3, addition of miR-199a precursor would up-regulate miR-199a expression in SW480 cells. In the various post-transfection time points, the CRC cell proliferation of miR-199a group was significantly reduced than the control group (P < 0.05) since the third day.

### MiR-199a transfection on apoptosis rate and cell-cycle of CRC cell

**Figure 4:**
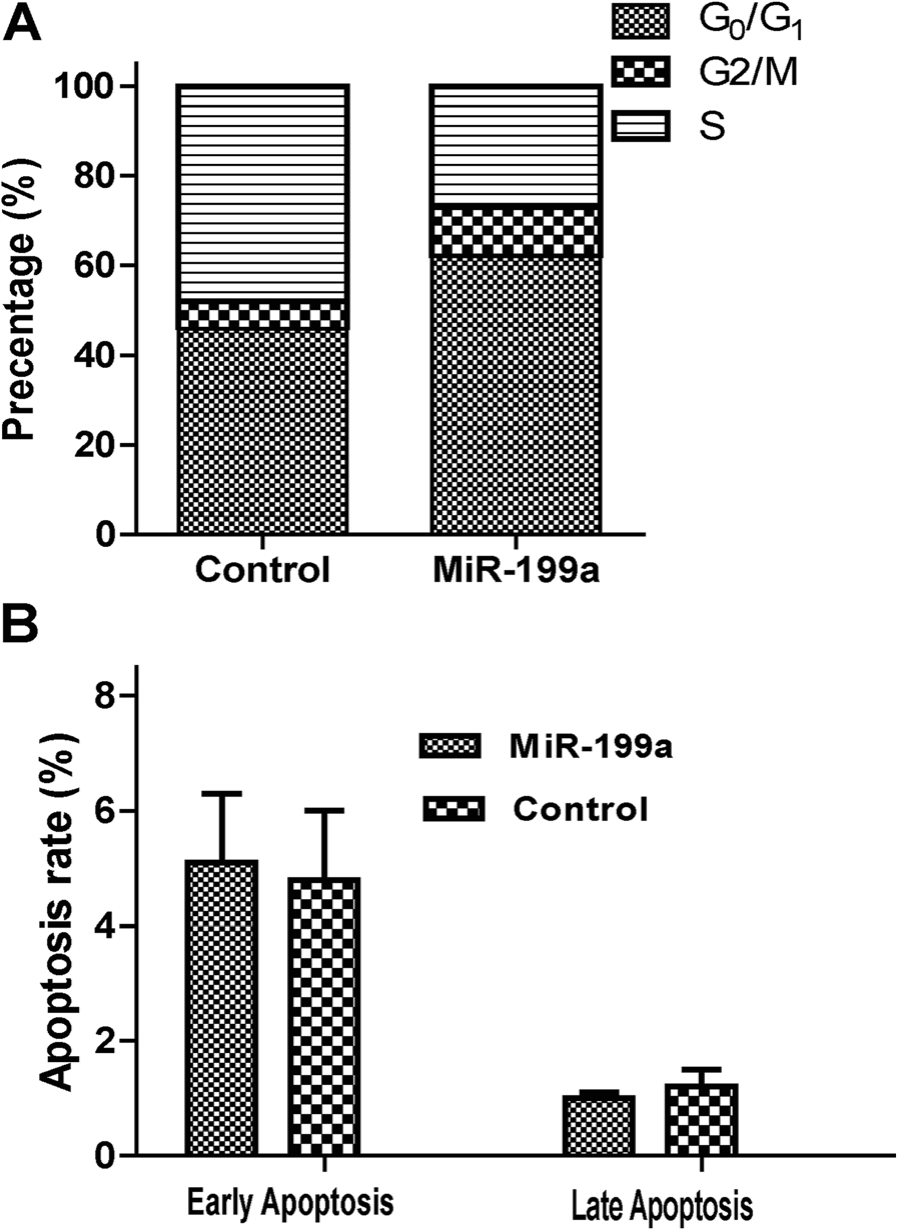
**A)** The cell cycles of CRC SW480 cells. **B)** The apoptosis rate of CRC SW480 cells. SW480 cells were plated and transfected with negative control or miRNA-199a mimics for 72 h. MiR-199a induced apoptosis of SW480 Cells. s. Flow cytometry analysis demonstrated that the percentage of the G_0_/G_1_, G_2_/M and S phase. The apoptosis rate was detected after transfection and tested with Annexin-V/PI.

We further investigated the effect of miR-199a on apoptosis of SW480 cells. SW480 cells were transfected with 100 nmol/L of miR-199a precursor mimics for 72 h. Flow cytometry analysis demonstrated that the percentage of the G_0_/G_1_, G_2_/M and S phase (43.2% for G_0_/G_1_ phase, 5.4% for G_2_/M phase and 51.4% for S phase) in control group. While the percentage of the G_0_/G_1_, G_2_/M and S phase (60.9% for G_0_/G_1_ phase, 8.8% for G_2_/M phase and 28.3% for S phase) in the miR-199a group were not significantly different with the control group (Figure 4A). To determine whether the SW480 cell growth regulation was attributed to apoptosis, we performed flow cytometric analysis of SW480 cells after transfection of miR-199a precursor mimics and the relative control minics. In miR-199a transfected SW480 group, the rates of early and late apoptosis rate are 5.2% and 0.9%. While the rates in control minics transfected cells were 4.8% and 1.1%, respectively (Figure 4B). No significant differences were detected in the apoptosis rate in both groups (P > 0.05).

### MiR-199a reduces migration and invasive phenotypes of SW480 cells

**Figure 5:**
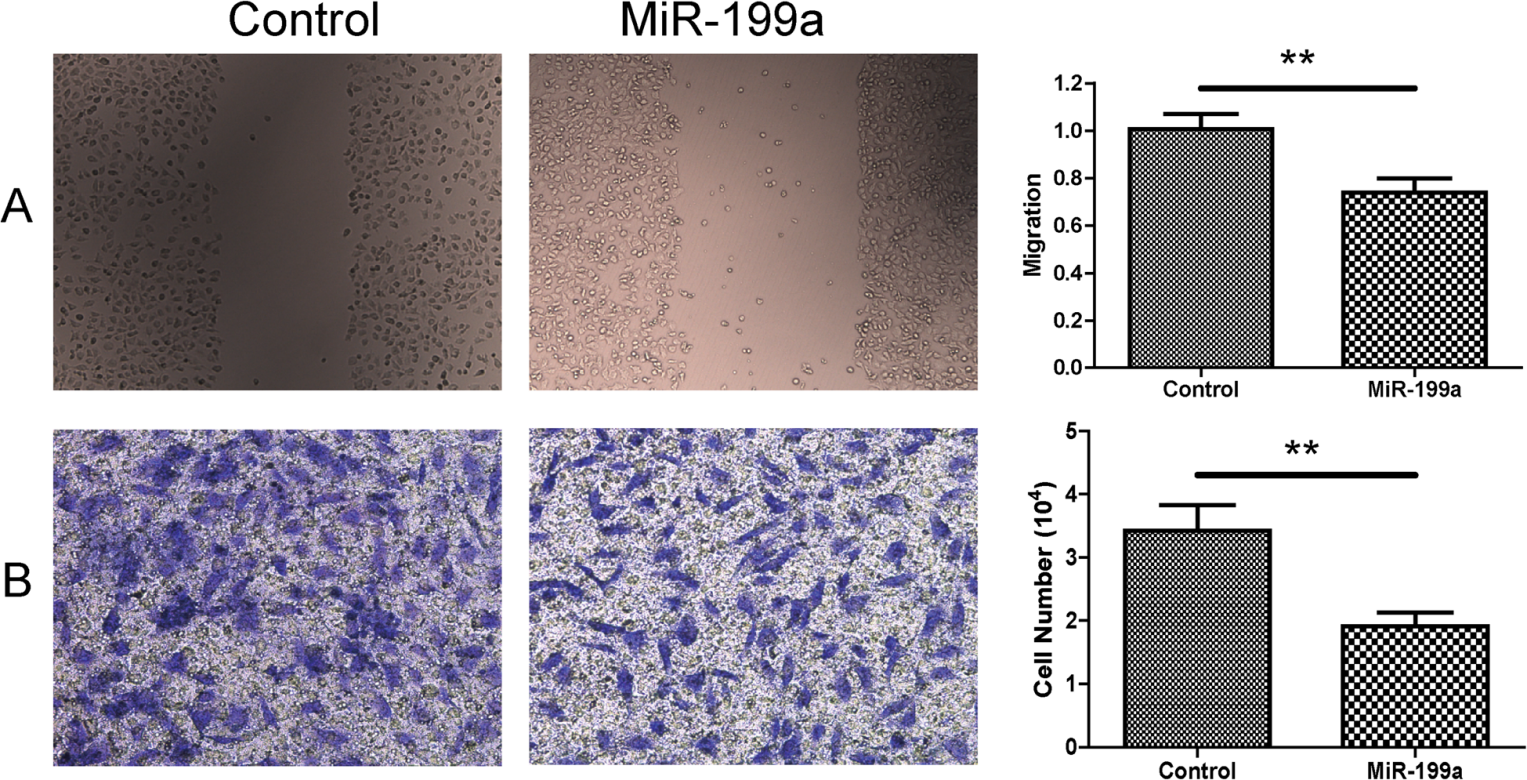
MiR-199a inhibited migration and invasion of SW480 cells. Cell migration and invasion ability was analyzed by scratch test and transwell tests after miR-199a or NC transfection. **A**: Representative images of scratch test transfected with miR-199a or NC. **B**: Quantification of the migratory cells by solubilization of crystal violet. Data represented mean ± SD, P < 0.01.

In the migration assay, we found that the SW480 cells transfected with miR-199a precursor mimics have a lower migration rate compared with the control group (0.77 ± 0.11 versus 1.00 ± 0.14, P < 0.01, Figure 5A). In the transwell assay for the invasive ability, the invasive cells per field of view through the porous transwells were significantly reduced in the miR-199a precursor mimics transfected cells compared to the control mimics transfected cells (1.95 ± 0.22 versus 3.34 ± 0.32, P < 0.01, Figure 5B).

### MiR-199a transfection was associated with decreased HIF-1α and VEGF

**Figure 6:**
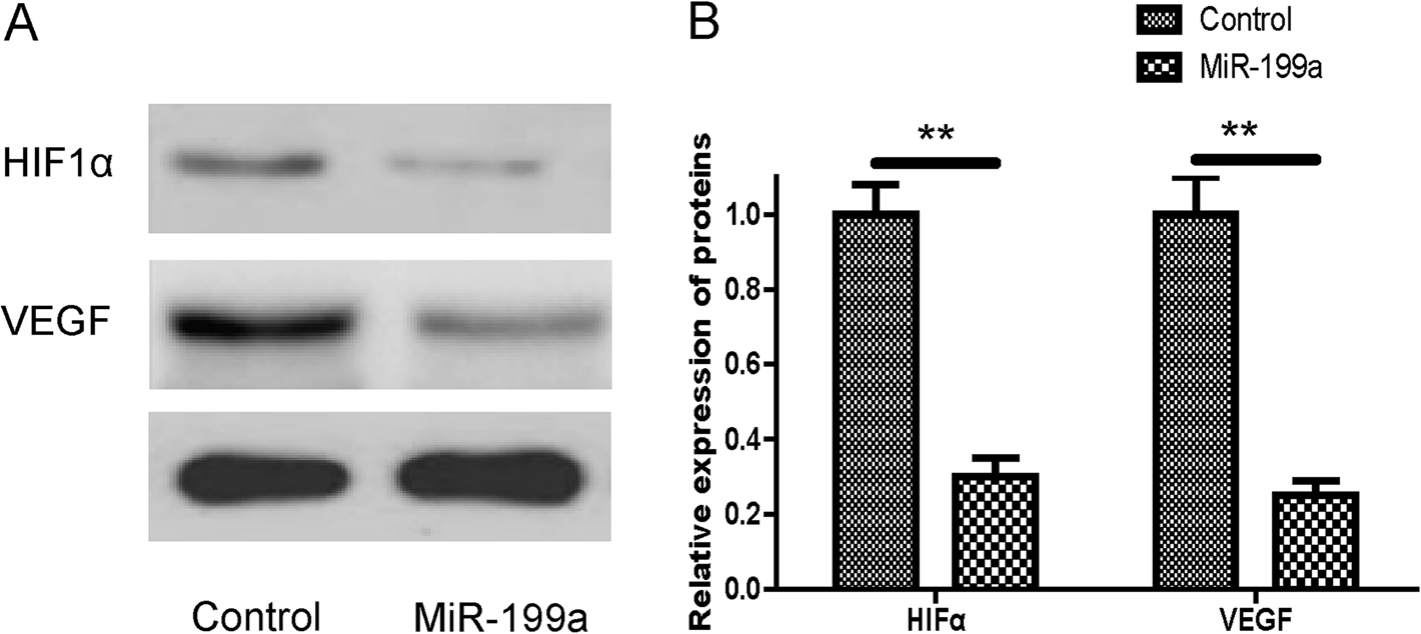
MiR-199a inhibited HIF-1α and VEGF expression in SW480 cells. **A**: Western blotting analysis of HIF-1α and VEGF protein level. The β-actin was used as a loading control. **B**: The expression level of HIF-1α and VEGF in SW480 cells, normalized by β-actin expression, P < 0.01.

We further determined the expression of HIF-1α/VEGF protein by western blot in CRC cells transfected with miR-199a precursor or control minics. To investigate the modification by miR-199a of the key HIF-1α/VEGF signaling proteins in human CRC cells, the expressions of HIF-1α and VEGF were determined by western-blot assay. As shown in Figure 6, miR-199a precursor mimics treatment was inversely correlated with decreased expression of HIF-1α and VEGF (Figure 6A and 6B, P < 0.01).

## Discussion

Emerging evidence has highlighted the crucial role of miRNA dysregulation on tumourigenesis of human CRC. MiRNA dysregulation modified cell proliferation, confers resistance to apoptosis, and enhances invasiveness and metastasis, through repressing the downstream target genes, and thus is involved in the initiation, progression, and metastasis of human tumors. In this study, we found that miR-199a down-regulation was associated with the CRC and metastasis incidence. Advanced study showed that miR-199a up-regulation would lead to decreased CRC proliferation, migration and invasion. However, no significant association of miR-199a treatment and apoptosis rate and cell-cycle were detected in this study. The mechanisms of miR-199a on the development of CRC showed that the anticarcinogenic effect of miR-199a might be produced through HIF-1α/VEGF pathway.

The discovery of miRNAs provided a powerful approach for exploring many complicated cellular biological functions in the tumorigenesis. In several published studies, miR-199a has been found to be in low expression level in kinds of cancers [14-17]. Up-regulation of miR-199a might be used as tumor repressor for kinds of cancer. In a previous study, Cheng *et al.* confirmed that miR-199a could target CD44 via a miR-199a-binding site in the 3′-UTR. The human miR-199a was cloned and transfected into ovarian CICs and the results found that CD44 mRNA and protein expression was significantly decreased in miR-199a-transfected ovarian CICs as compared with miR-199a mutant-transfected and untransfected cells. Cell cycle analysis, the colony formation assay and the transwell migration assay indicated that miR-199a significantly affected cell cycle regulation and suppressed the proliferation and invasive capacity of ovarian CICs *in vitro* [18]. Tsukigi M *et al.* [19] conducted an independent study and they reportedshow that re-expression of miR-199a downregulated GSK-3β and suppresses cancer cell growth. The results demonstrate low miR-199a expression as a feature of advanced renal cell carcinoma, identify miR-199a as a negative regulator of GSK-3β, and suggest re-expression of pre-miR-199a as a new potential treatment of renal cell carcinoma. For the CRC, decreased miR-199a expression was detected compared with the controls. Hu *et al.* reported that overexpression of miR-199a would result in reduced colony formation, invasive and migratory capabilities of different human CRC cell lines [20]. Through the dual luciferase reporter assay, it was also found that overexpression of miR-199a-5p led to decrease DDR1, MMP2, N-cadherin and vimentin expression and increased E-cadherin expression through binding to their 3′-UTR sites.

In previous study, the tissue hypoxia induces reprogramming of cell metabolism and may result in normal cell transformation and cancer progression. HIF-1α, the key transcription factor, plays an important role in CRC development and progression [21]. VEGF is over-expressied in CRC cells and plays a critical role in angiopoiesis and cell proliferation, making it a potential target for cancer therapy. A recognized cancer suppressor, phosphatase and tensin homologue (PTEN), has been reported to be associated with the development of CRC. In a previous study, PTEN was reported to have a correlation with VEGF expression via HIF-1α, and the PI3K/mTOR pathways [22]. In a retrospective study, it evaluated the HIF-1α expression by immunohistochemical staining and analyzed its association with several clinicopathological characteristics. It showed a significant correlation was also observed between the expressions of HIF-1α and VEGF in liver metastases and primary CRC [23]. Nagaraju GP *et al.* reported that ganetespib could work as a potential anti-cancer agent and it effected through HIF-1α/VEGF pathway [21]. The results provided potential eligible drug detection for the CRC.

MiR-199a has been reported to be a potential inhibitor of HIF-1α/VEGF pathway. Joshi *et al.* reported that miR-199a targets the 3′-UTR of HIF-1α and HIF-2α. Decreased miR-199a expression in hypoxia increased HIF levels. Exogenous expression of miR-199a decreased HIF, cell migration, and metastasis of ovarian cancer cells [24]. In an *in vivo* and *in vitro* study, up-expression of miR-199a and miR-125b inhibited tumor-induced angiogenesis associated with the decrease of HIF-1α and VEGF expression in ovarian cancer cells. Moreover, the levels of miR-199a and miR-125b were negatively correlated with VEGF mRNA levels in ovarian tissues. We further showed that direct targets of miR-199a and miR-125b HER2 and HER3 were functionally relevant. Forced expression of HER2 and HER3 rescued miR-199a- and miR-125b-inhibiting angiogenesis responses and Akt/p70S6K1/HIF-1α pathway [25]. Compared to parental cells or cells transfected with a control vector, the over-expression of microRNA-199a in the hepatocellular carcinoma cell lines stably was showed to reduce cell proliferation *in vitro* and *in vivo*. Advanced study revealed the regulation of miR-199a on 3′-UTR of HIF-1α. Further investigation confirmed that miR-199a significantly reduced the endogenous protein level of HIF-1α in hypoxia. MiR-199a inhibits cell proliferation *in vitro* and *in vivo* partly through down-regulation of HIF-1α in human hepatocellular carcinoma [26].

## Conclusion

In conclusion, we conducted detailed analyses on the effect of miR-199a on the CRC cell line. Through the analyses on the miR-199a on the biological behaviors of CRC cell line, we found that miR-199a would reduce the proliferation, migration and invasion. However, overexpression of miR-199a on the apoptosis rate and cell cycles showed no significant results. The potential functionary mechanism of miR-199a might through HIF-1α/VEGF pathway.

## Abbreviations

CRC: Colorectal cancer
HIF-1α: Hypoxia-inducible factor 1α
VEGF: Vascular endothelial growth factor
miRNA: Micro RNA
PDK1: Pyruvate dehydrogenase lipoamide kinase isozyme 1
MTT: 3-(4,5-dimethylthiazol-2-yl)-2,5- diphenyl-2H-tetrazolium bromide
DMSO: Dimethyl Sulfoxide
PBS: Phosphate-buffered saline
FBS: Fetal calf serum
PTEN: Phosphatase and tensin homologue
